# Extraciliary OFD1 Is Involved in Melanocyte Survival through Cell Adhesion to ECM via Paxillin

**DOI:** 10.3390/ijms242417528

**Published:** 2023-12-15

**Authors:** Nan-Hyung Kim, Chang Hoon Lee, Ai-Young Lee

**Affiliations:** 1 Department of Dermatology, Dongguk University Ilsan Hospital, 814 Siksa-dong, Ilsandong-gu, Goyang-si 10326, Republic of Korea; 2 College of Pharmacy, Dongguk University, Seoul 04620, Republic of Korea; uatheone@dongguk.edu

**Keywords:** OFD1, vitiligo, paxillin, ECM adhesion, nonciliary mechanism, melanocyte apoptosis

## Abstract

Primary cilia play a significant role in influencing cell fate, including apoptosis in multiple cell types. In the lesional epidermis of vitiligo patients, a reduced number of ciliated cells was observed. Our study also revealed a downregulation of oral–facial digital syndrome type 1 (OFD1) in the affected skin of vitiligo patients. However, it remains unknown whether primary cilia are involved in the control of melanocyte apoptosis. While both *intraflagellar transport 88 (IFT88)* and *retinitis pigmentosa GTPase regulator-interacting protein-1 like (RPGRIP1L)* are associated with ciliogenesis in melanocytes, only the knockdown of *OFD1*, but not *IFT88* and *RPGRIP1L*, resulted in increased melanocyte apoptosis. *OFD1* knockdown led to a decrease in the expression of proteins involved in cell–extracellular matrix (ECM) interactions, including paxillin. The OFD1 amino acid residues 601-1012 interacted with paxillin, while the amino acid residues 1-601 were associated with ciliogenesis, suggesting that the OFD1 domains responsible for paxillin binding are distinct from those involved in ciliogenesis. *OFD1* knockdown, but not *IFT88* knockdown, inhibited melanocyte adhesion to the ECM, a defect that was restored by paxillin overexpression. In summary, our findings indicate that the downregulation of OFD1 induces melanocyte apoptosis, independent of any impairment in ciliogenesis, by reducing melanocyte adhesion to the ECM via paxillin.

## 1. Introduction

Melanocytes, specialized cells responsible for melanin synthesis, are vital for the survival of all living organisms on Earth. The chronic and progressive loss or death of melanocytes leads to vitiligo, the most common yet challenging-to-treat depigmentation disorder characterized by white macules and patches on the skin and hair [1]. Vitiligo is a complex, multifactorial, and polygenic disease. While growing evidence suggests that oxidative stress linked to the immune system plays a critical role in the pathogenesis of vitiligo [2], the precise mechanisms underlying melanocyte apoptosis and the development of vitiligo remain to be elucidated.

Primary cilia extend from the apical surface like antennas, serving as sensory organelles for extracellular signals, and influencing cell fate in various ways. The signal transduction is carried out via various pathways including Hedgehog and Wnt [3,4,5,6,7,8]. Dysfunctional cilia have been associated with numerous pathologies, including recognized ciliopathies [9]. These organelles are present in nearly all cell types, including melanocytes and basal keratinocytes, within the normal epidermis [10]. The loss of primary cilia on melanocytes has been proposed as a top predictive feature in the diagnosis of melanoma [11]. Our preliminary findings indicate a decrease in the number of melanocytes and keratinocytes with primary cilia in lesional epidermis compared to nonlesional epidermis in vitiligo patients (Figure S1). This suggests that primary cilia may play a role in melanocyte signaling and the functioning of melanocytes, although the specific involvement of primary cilia in melanocyte apoptosis remains unidentified.

To explore the role of primary cilia in melanocyte apoptosis further, we conducted a microarray analysis of nonlesional and lesional epidermis from two patients with vitiligo to identify differentially expressed genes (DEGs) related to ciliogenesis. Numerous genes are known to be involved in ciliogenesis and ciliary functions [10,12]. In our analysis, we observed a significant downregulation of *OFD1* among the DEGs, which overlapped with known ciliogenesis genes). Oral–facial–digital syndrome type 1 (OFD1), localized at the basal body, is involved in the assembly of primary cilia [13,14]. OFD1 syndrome is a well-known ciliopathy caused by mutations of *OFD1* [15]. Notably, OFD1 syndrome does not typically manifest vitiligo as a clinical characteristic. Recent research has revealed that OFD1 is a highly pleiotropic protein. OFD1 is necessary for the formation of primary cilia and left-right asymmetry establishment. However, OFD1 can also be involved in other tasks, such as control of centrioles length and distal structure, chromatin remodeling, regulation of cellular protein content, protein quality balance, cell cycle progression, and selective autophagy, via nonciliary activities [16]. A neuroprotective function of OFD1 in the rat retina has also been demonstrated by protecting photoreceptors from oxidative stress and apoptosis via a nonciliary mechanism [17].

In this study, we investigated the role of OFD1 in vitiligo, particularly focusing on melanocyte apoptosis. The impact of OFD1 on melanocyte apoptosis can be assessed by comparing with other genes involved in ciliogenesis and ciliary function. Among numerous ciliogenic genes, *intraflagellar transport (IFT)* plays a critical role in the elongation of the cilium axoneme for ciliary assembly as a bidirectional transport system [18]. *Retinitis pigmentosa GTPase regulator-interacting protein-1 like (RPGRIP1L)* is another basal body gene involved in ciliogenesis [19], like *OFD1*. Thus, we chose these two genes alongside *OFD1* for the assessment.

## 2. Results

### 2.1. OFD1 Is Downregulated in Lesional Epidermis of Patients with Vitiligo

**Figure 1:**
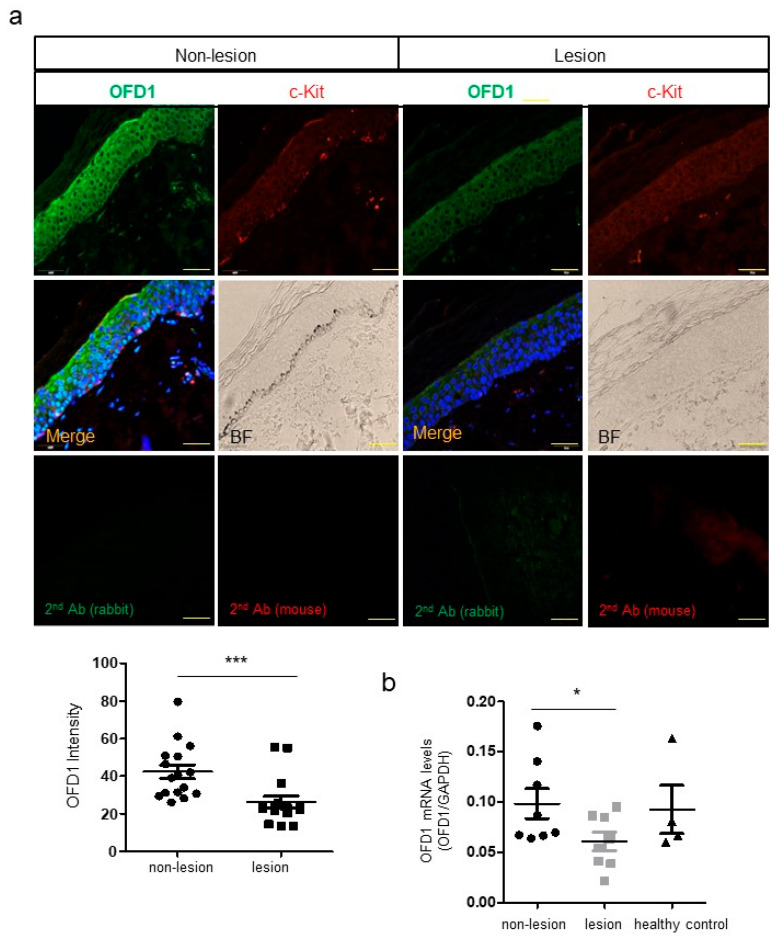
OFD1 is downregulated in the lesional epidermis of patients with vitiligo. (**a**) Representative immunofluorescent microscopy images using anti-OFD1 and anti-c-KIT antibodies in lesional and nonlesional skin biopsy samples obtained from eight patients with vitiligo (bar = 0.05 mm). The nuclei were counterstained with Hoechst 33258. The intensities were quantified at two randomized high-power fields/each specimen using ImageJ software version 1.54d. (**b**) Real-time PCR for the relative mRNA levels of OFD1 in the lesional and nonlesional skin specimens obtained from eight patients with vitiligo and four healthy individuals. GAPDH was used as an internal control. The data from patients’ skin specimens in each graph represent the mean ± SEMs. * *p* < 0.05 and *** *p* < 0.001.

Immunofluorescence microscopy revealed reduced relative expression ratios of OFD1 protein in the lesional epidermis, particularly in the lower portion. Notably, c-KIT-positive melanocytes in the nonlesional epidermis were also stained with an anti-OFD1 antibody (Figure 1a). Real-time PCR data indicated that the relative expression levels of OFD1 mRNA were reduced in the lesional epidermis compared to those in the nonlesional epidermis and healthy control skin (Figure 1b).

### 2.2. OFD1 Is Involved in Ciliogenesis and Apoptosis of Keratinocytes and Melanocytes

**Figure 2:**
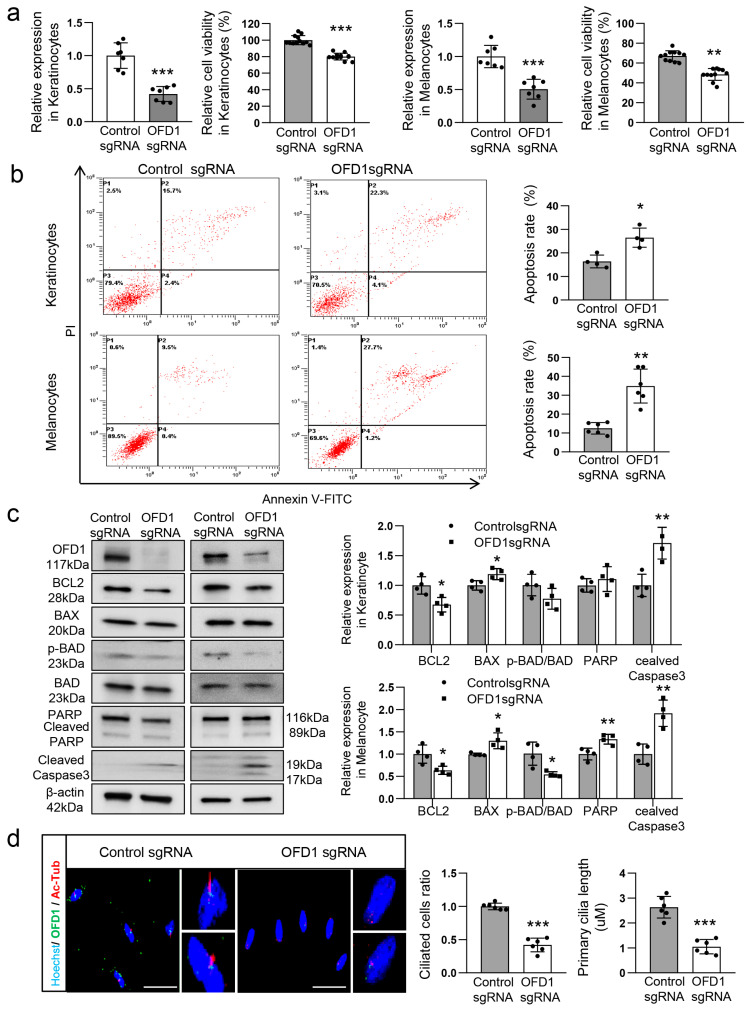
OFD1 is involved in the ciliogenesis and cell survival of keratinocytes and melanocytes. (**a**–**c**) MTT assay combined with Western blot analysis of the relative levels of OFD1 protein (**a**), FACS analysis (**b**), and Western blot analysis of BCL2, BAX, phospho-BAD, BAD, cleaved PARP, and cleaved caspase-3 (**c**) in primary cultured adult human keratinocytes and melanocytes following OFD1 knockdown (OFD1 sgRNA) or control RNA knockdown (control sgRNA) for 48 h. GAPDH and β-actin were used as internal controls for real-time PCR and Western blot analysis, respectively. (**d**) Representative confocal microscopy equipped with NIS-Elements AR 3.2 program to measure the length of primary cilia using anti-OFD1 and antiacetylated α-tubulin (Ac-Tub) antibodies in cultured melanocytes with or without OFD1 knockdown (bar = 0.025 mm). The nuclei for confocal microscopy were counterstained with Hoechst 33258. The data from cultured keratinocytes and melanocytes in each graph represent the mean ± SDs of six independent experiments. * *p* < 0.05, ** *p* < 0.01, and *** *p* < 0.001.

Following OFD1 downregulation, ciliogenesis was observed to be reduced in the lesional epidermis (Figure 1a). Vitiligo is associated with melanocyte death, which can occur passively through neighboring keratinocytes [20,21]. Therefore, we examined the role of OFD1 in ciliogenesis and cell survival using primary cultured adult human keratinocytes and melanocytes, both with and without OFD1 knockdown. The MTT assay showed a decrease in the ratio of viable cells to total cells in keratinocytes and melanocytes following OFD1 knockdown using clustered regularly interspaced short palindromic repeat (CRISPR) technology (Figure 2a). Flow cytometric analysis demonstrated an increase in annexin V-positive/propidium iodide (PI)-negative and double-positive proportions in both keratinocytes and melanocytes after OFD1 knockdown (Figure 2b). OFD1 knockdown resulted in decreased relative ratios of BCL2: BAX and phosphorylated BCL2-associated death (pBAD) protein levels but increased cleaved poly-(ADP-ribose) polymerase (PARP) and cleaved caspase-3 protein levels (Figure 2c). The analysis of cilia length performed by capturing series of z-stack confocal images, utilizing double staining with anti-OFD1 and antiacetylated α-tubulin antibodies and equipped with the NIS-Elements AR 3.2 program, revealed shorter or unrecognizable primary cilia in melanocytes following OFD1 knockdown compared to control (Figure 2d).

### 2.3. Knockdown of IFT88 or RPGRIP1L Impairs Ciliogenesis without Increasing Melanocyte Apoptosis

**Figure 3:**
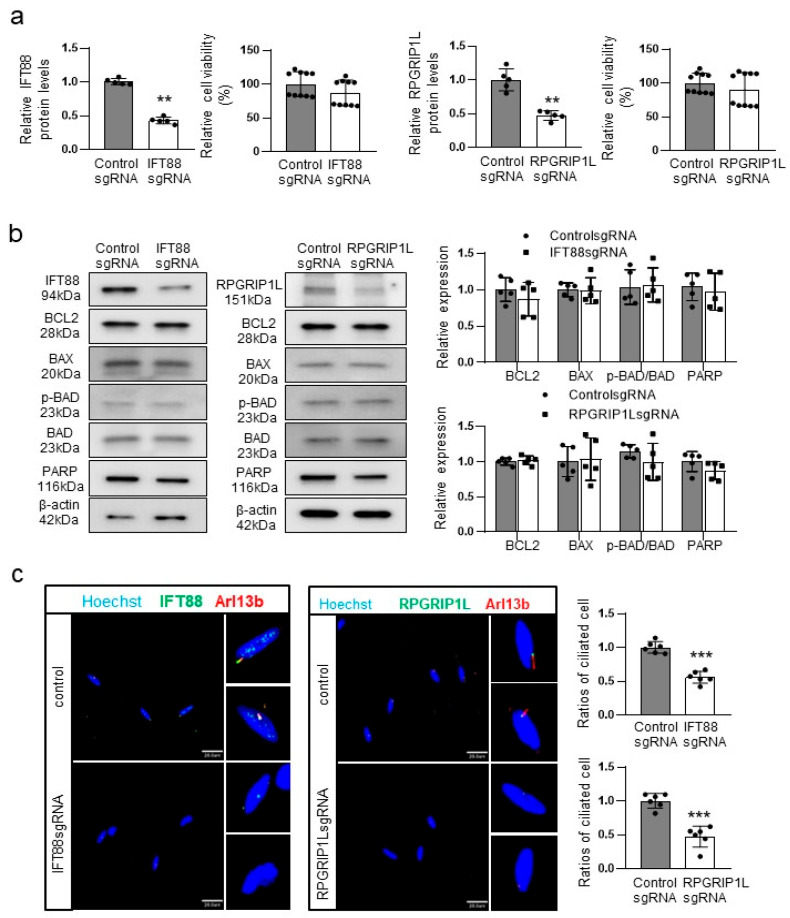
Knockdown of IFT88 or RPGRIP1L impairs ciliogenesis without increasing melanocyte apoptosis. (**a**,**b**) MTT assay combined with Western blot analysis of the relative levels of IFT88 and RPGRIP1L proteins (**a**) and Western blot analysis of BCL2, BAX, phospho-BAD, BAD, and cleaved PARP (**b**) in cultured melanocytes with or without IFT88 or RPGRIP1L knockdown for 48 h. β-actin was used as an internal control for the Western blot analysis. (**c**) Representative confocal microscopy using anti-IFT88 or anti-RPGRIP1L and anti-ARL13B antibodies to determine the relative number of cells containing cilia to the total number of cells (bar = 0.02 mm). The nuclei for confocal microscopy were counterstained with Hoechst 33258. The data from cultured melanocytes in each graph represent the mean ± SDs of six independent experiments. ** *p* < 0.01 and *** *p* < 0.001.

OFD1 knockdown was associated with reduced cell survival and ciliogenesis (Figure 2a–d). Consequently, we examined the effects of knocking down other ciliogenesis-related genes, IFT88 or RPGRIP1L, on cellular apoptosis and ciliogenesis using cultured melanocytes. Knockdown of IFT88 or RPGRIP1L, which led to a reduction of the corresponding proteins by more than 50%, did not decrease the relative ratios of viable cells to total cells (Figure 3a). Additionally, there were no significant changes in the relative ratios of BCL2, BAX, pBAD, and cleaved PARP protein levels due to IFT88 or RPGRIP1L knockdown (Figure 3b). However, the analysis of cilia length performed by capturing a series of z-stack confocal images showed that knockdown of IFT88 or RPGRIP1L resulted in shorter primary cilia in melanocytes (Figure 3c).

### 2.4. Knockdown of OFD1, IFT88, or RPGRIP1L Reduces Hedgehog Signaling Pathways in Melanocytes

**Figure 4:**
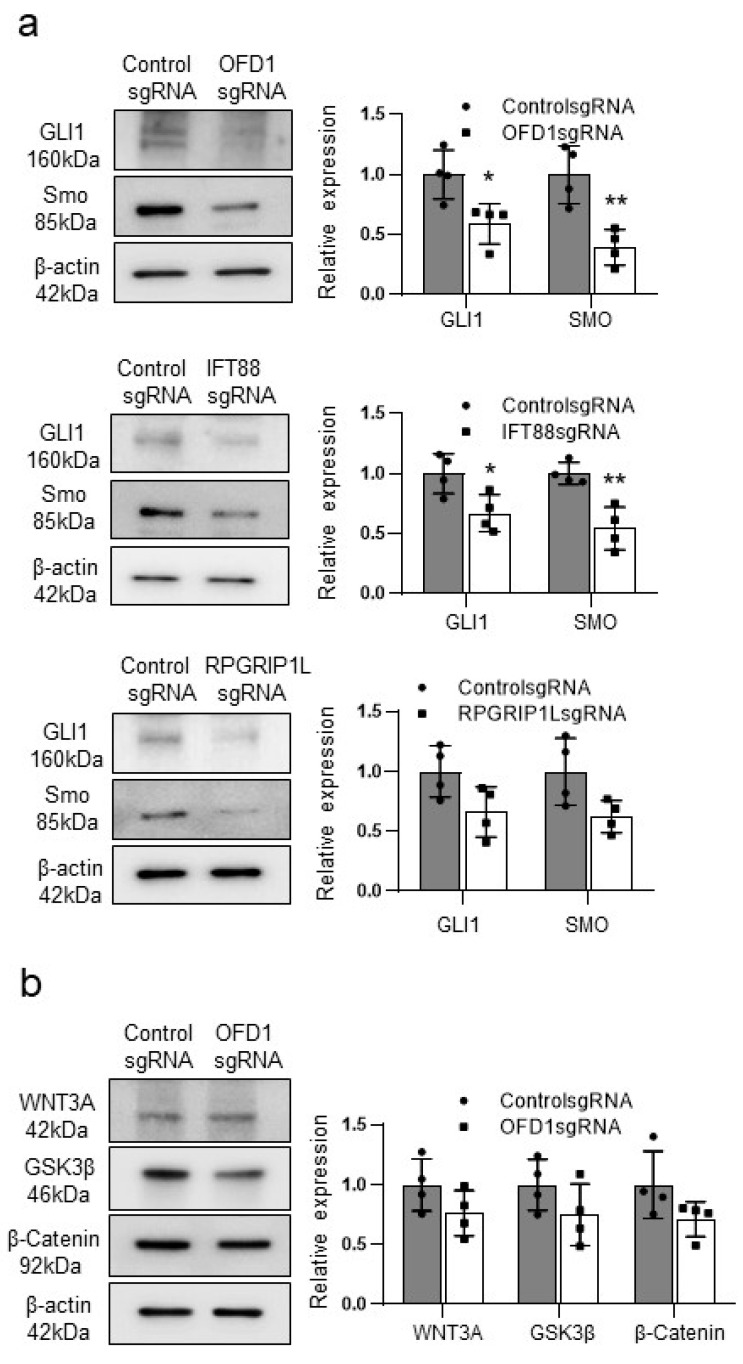
Knockdown of OFD1 as well as IFT88 or RPGRIP1L reduces hedgehog signaling pathways in melanocytes. (**a**) Western blot analysis of the relative ratios of PTCH1, GLI1, and Smo protein levels in cultured melanocytes with or without OFD1, IFT88, or RPGRIP1L knockdown. (**b**) Western blot analysis of the relative ratios of WNT3a, GSK-3β, and β-catenin in cultured melanocytes with or without OFD1 knockdown. Β-actin was used as an internal control. The data from cultured melanocytes in each graph represent the mean ± SDs of six independent experiments. * *p* < 0.05 and ** *p* < 0.01.

In contrast to OFD1 (Figure 2a–c), knockdown of IFT88 or RPGRIP1L did not lead to an increase in melanocyte apoptosis (Figure 3a,b). To investigate the underlying mechanism, we explored the basic signaling pathways associated with primary cilia [2,3,4,5,6] in cultured melanocytes following OFD1 knockdown. The results of OFD1 knockdown were compared with those of IFT88 or RPGRIP1L knockdown, if necessary. Western blot analysis revealed decreased relative levels of Hedgehog signaling proteins, including patched1 (PTCH1), glioma-associated oncogene homolog1 (GLI1), and smoothened (Smo), in melanocytes with OFD1 knockdown (Figure 4a). However, the relative levels of Wnt signaling proteins, such as WNT3A, GSK3β, and β-catenin, remained largely unchanged following OFD1 knockdown (Figure 4b). Similarly, the relative levels of Hedgehog signaling proteins were reduced in melanocytes following IFT88 or RPGRIP1L knockdown (Figure 4a).

### 2.5. OFD1 Knockdown Reduces Interactions with the Extracellular Matrix (ECM) via Paxillin

**Figure 5:**
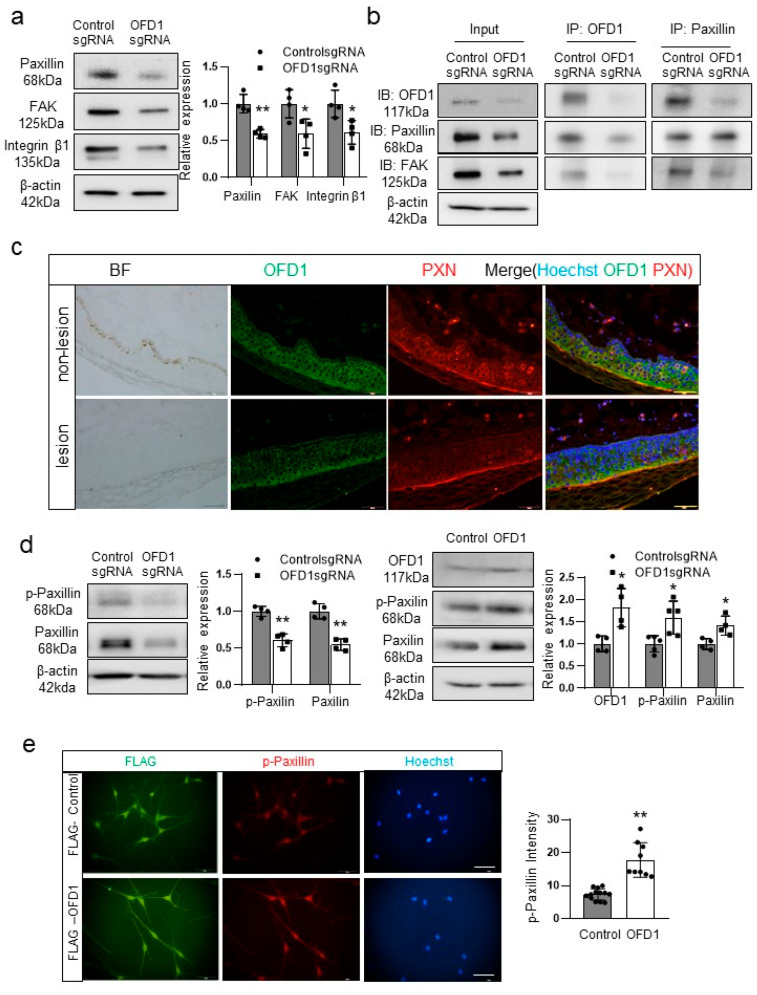
OFD1 knockdown reduces interactions with the ECM via paxillin. (**a**,**b**) Western blot analysis of the relative ratios of paxillin, FAK, and integrin β1 (**a**) and immunoprecipitation using anti-OFD1 or antipaxillin antibody (**b**) in cultured melanocytes with or without OFD1 knockdown. (**c**) Representative immunofluorescent microscopy using anti-OFD1 and antipaxillin antibodies in lesional and nonlesional skin biopsy samples from eight patients with vitiligo (bar = 0.05 mm). (**d**) Western blot analysis of the relative ratios of phosphorylated paxillin in cultured melanocytes with or without OFD1 knockdown or OFD1 overexpression. (**e**) Representative immunofluorescent microscopy images using anti-FLAG and antiphosphopaxillin antibodies in melanocytes transfected with FLAG alone or the chimeric FLAG-OFD1 vector (bar = 0.05 mm). β-actin was used as an internal control in the Western blot analysis. The nuclei for immunofluorescent microscopy analysis were counterstained with Hoechst 33258. The data from cultured melanocytes in each graph represent the mean ± SDs of six independent experiments. * *p* < 0.05 and ** *p* < 0.01.

Given the similarity in the reduction of Hedgehog signaling proteins by OFD1, IFT88, and RPGRIP1L knockdown (Figure 4a), and the absence of significant changes in Wnt signaling proteins (Figure 4b), no significant role of these signaling pathways was suggested in melanocyte apoptosis induced by OFD1 knockdown. Thus, based on the interaction between primary cilia and the ECM [22,23], the proteins involved in cell–ECM interactions were examined. Western blot analyses indicated that the relative levels of paxillin, focal adhesion kinase (FAK), and integrin β1 were reduced in OFD1-knockdown melanocytes (Figure 5a). The immunoprecipitation assay suggested interactions between OFD1 and paxillin, and between paxillin and FAK (Figure 5b). Downregulation of paxillin was also detected in the lesional epidermis of vitiligo patients with OFD1 downregulation (Figure 5c). OFD1 knockdown reduced paxillin phosphorylation at residue 118, which regulates focal adhesions [24,25], while OFD1 overexpression increased paxillin phosphorylation (Figure 5d). Transfection with the chimeric FLAG-OFD1 vector followed by immunofluorescence microscopy revealed that OFD1 overexpression increased the expression levels of phosphorylated paxillin (Figure 5e).

### 2.6. OFD1 Domains Involved in Paxillin Binding Differ from Those Participating in Ciliogenesis

**Figure 6:**
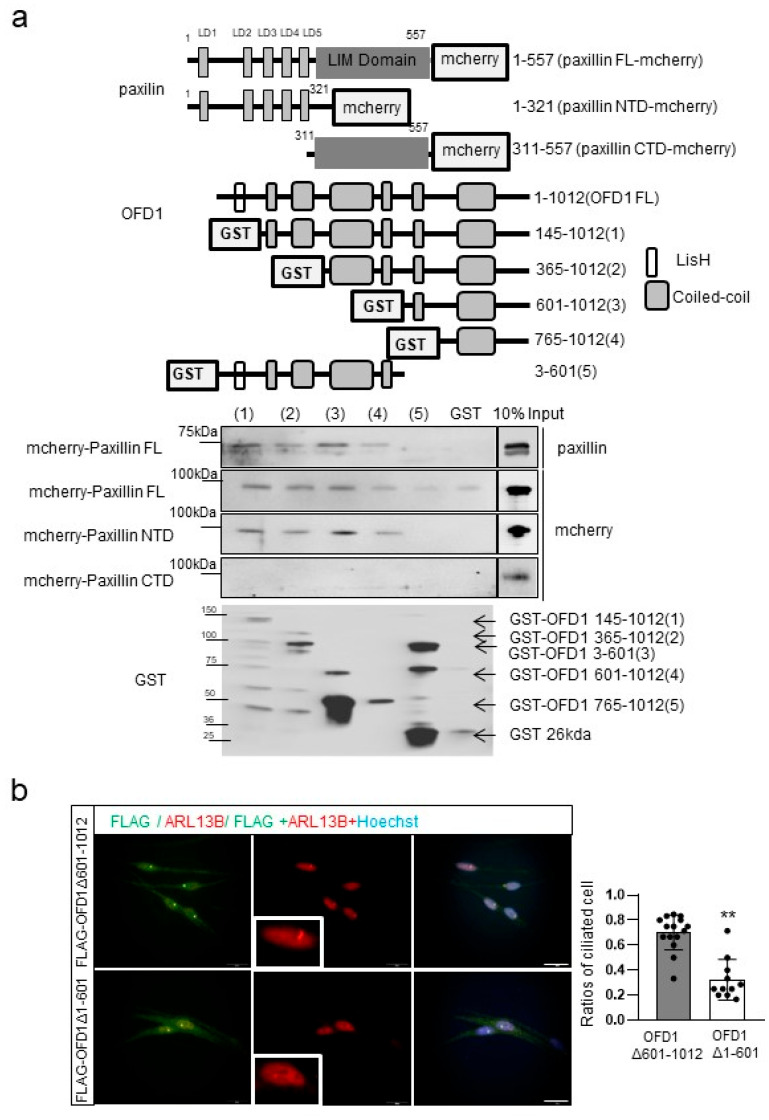
C-terminal domain of OFD1 binds to the LD domains of paxillin. (**a**) Western blot analysis of purified GST or GST-tagged OFD1 fragments (1-5) incubated with lysates containing full-length (FL), N-terminal domain (NTD), or C-terminal domain (CTD) of PXN-transfected 293T cells. (**b**) Representative immunofluorescent microscopy images of anti-ARL13B in melanocytes transfected with chimeric FLAG-OFD1 with deleted amino acid residues 601-1012 (OFD1Δ—601-1012) or 1-601 (OFD1Δ1-601) (bar = 0.02 mm). The data from cultured melanocytes in each graph represent the mean ± SDs of six independent experiments. ** *p* < 0.01.

The difference in the impact of ciliogenesis and apoptosis in melanocytes following OFD1, IFT88, or RPGRIP1L knockdown suggests that the domains responsible for ciliogenesis in OFD1 may differ from those responsible for paxillin binding. The glutathione-S-transferase (GST) pull-down assay indicated that OFD1 amino acid residues 145-1012, OFD1 —365-1012, OFD1 601-1012, and OFD1 765-1012, but not the OFD1 3-601 construct [26], interacted with the N-terminal-containing LD domains rather than the C-terminal-containing LIM domains of paxillin, fused with an mCherry fluorescent reporter protein (Figure 6a). On the other hand, immunofluorescence staining showed that melanocytes transfected with chimeric FLAG-OFD1 with amino acid residues —1-601 deleted (Δ—1-601) exhibited shorter or less recognizable primary cilia compared to those transfected with FLAG-OFD1 with residues —601-1012 deleted (Δ—601-1012) (Figure 6b).

### 2.7. Downregulation of Paxillin by OFD1 Knockdown Inhibits Melanocyte Adhesion to the ECM

**Figure 7:**
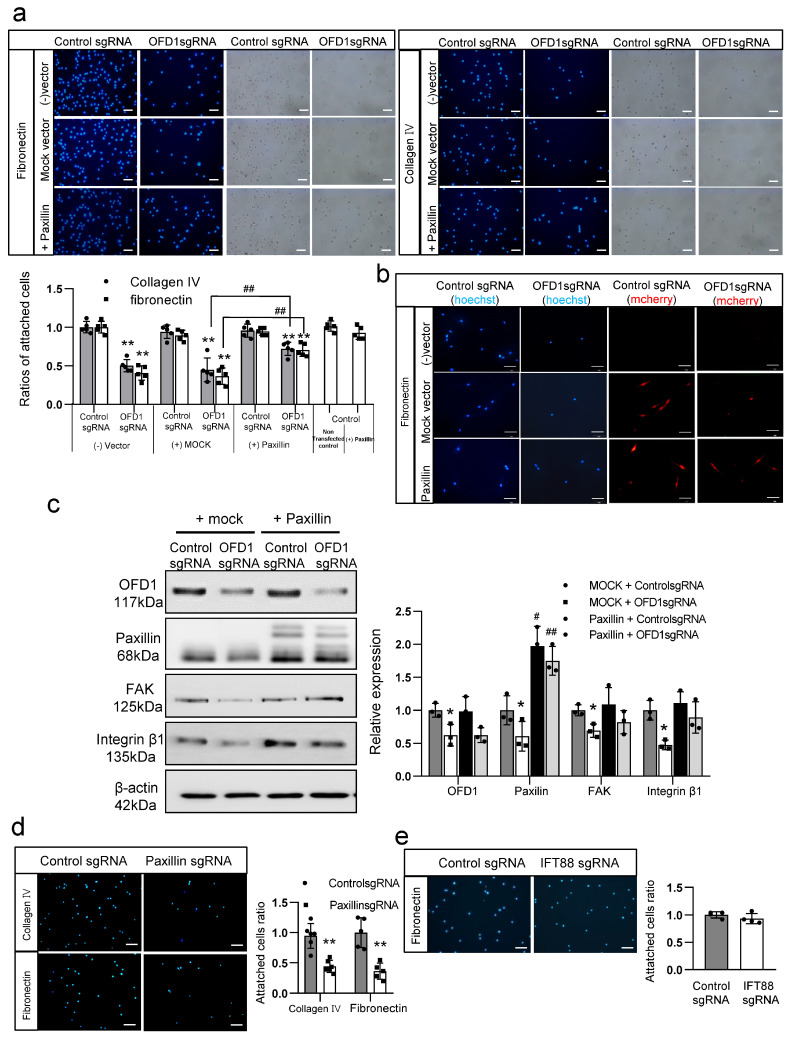
Downregulation of paxillin by OFD1 knockdown inhibits melanocyte adhesion to the ECM. (**a**,**b**) Adhesion assays performed on fibronectin or type IV collagen-coated culture dishes with immunofluorescent microscopy for nuclei (**a**) or mCherry (**b**) using cultured melanocytes with control RNA (control sgRNA) or OFD1 knockdown (OFD1 sgRNA) in the presence (paxillin) or absence (mock vector) of mCherry-fused PXN overexpression or nontransfected control (−vector), and cells with mCherry-fused PXN overexpression (bar = 0.05 mm). (**c**) Western blot analysis of the relative ratios of paxillin, FAK, and integrin β1 in cultured melanocytes with or without OFD1 knockdown in the presence or absence of PXN overexpression. (**d**,**e**) Adhesion assay performed on fibronectin- or type IV collagen-coated culture dishes using cultured melanocytes with control sgRNA or PXN knockdown (PXN sgRNA) (**d**) and cells with IFT88 knockdown (IFT88 sgRNA) (**e**). β-actin was used as an internal control in the Western blot analysis. The nuclei for immunofluorescence staining were stained with Hoechst 33,258 (bar = 0.05 mm). The data from cultured melanocytes in each graph represent the mean ± SDs of six independent experiments. * *p* < 0.05 and ** *p* < 0.01 vs. control, # *p* < 0.05 and ## *p* < 0.01 vs. sample without PXN overexpression.

To elucidate the functional role of OFD1 in binding to paxillin, we performed an ECM adhesion assay using melanocytes with or without OFD1 knockdown or PXN (paxillin gene) overexpression, with or without PXN knockdown. These results indicated that OFD1 knockdown reduced the number of DAPI-positive nuclei adhering to fibronectin or type IV collagen (Figure 7a). No significant reduction in the number of adhered nuclei was observed after silencing of the control gene (Figure 7a). The reduced number of adhered nuclei to the ECM components by OFD1 knockdown was significantly restored by PXN overexpression, although PXN overexpression alone did not increase the number of adhered nuclei compared to the nontransfected control (Figure 7a). Immunofluorescence microscopy was performed using mCherry, the most widely used monomeric red fluorescent protein for live cell imaging [27], as a fusion protein. Following mCherry-fused PXN overexpression in melanocytes, with or without OFD1 knockdown, the adhered cells were observed to be paxillin-positive (Figure 7b). The reduced levels of paxillin, FAK, and integrin β1 in melanocytes following OFD1 knockdown were restored by PXN overexpression (Figure 7c). PXN knockdown reduced the number of nuclei attached to ECM components (Figure 7d). IFT88 knockdown did not reduce the number of nuclei that adhered to fibronectin (Figure 7e).

## 3. Discussion

The findings presented in this study strongly support the downregulation of OFD1 in the lesional epidermis of vitiligo patients. Immunofluorescence microscopy results (Figure 1a) and real-time PCR data (Figure 1b) provide confirmation of this downregulation. *OFD1* knockdown, which was achieved using CRISPR technology to minimize off-target effects of siRNA, resulting in increased apoptosis in both melanocytes and keratinocytes (Figure 2a–c), suggesting that OFD1 downregulation may play a significant role in vitiligo. Additionally, confocal microscopy revealed that the primary cilia in cultured melanocytes were shorter or unrecognizable following *OFD1* knockdown (Figure 2d), confirming the role of OFD1 in ciliogenesis, consistent with previous studies [10,13,28]. The observed increase in melanocyte apoptosis and impaired ciliogenesis following *OFD1* knockdown raises questions about the potential association between ciliogenesis and cellular apoptosis. However, no increase in apoptosis was observed following the knockdown of other ciliogenic genes, *IFT88* and *RPGRIP1L* (Figure 3a,b), despite impaired ciliogenesis in melanocytes (Figure 3c). This disconnect suggests that the relationship between primary cilia formation and melanocyte survival leading to vitiligo is more complex and merits further investigation. Among the basic signaling pathways associated with primary cilia [2,3,4,5,6], *OFD1* knockdown did not significantly affect the protein levels involved in the Wnt signaling pathway (Figure 4b). On the other hand, *OFD1* knockdown did reduce the levels involved of Hedgehog signaling pathways (Figure 4a). However, a similar reduction of proteins involved in Hedgehog signaling pathways was also observed following *IFT88* and *RPGRIP1L* knockdown (Figure 4a). This suggests that there may not be a direct connection between these signaling pathways and the OFD1-induced apoptosis.

The enrichment of integrins and ECM receptors in primary cilia has been reported [29,30]. Disruption of cilia-based signaling has been associated with altered integrin activity, ECM interactions, and matrix metalloproteinases (MMPs), which can lead to fibrosis [23]. This indicates a potential interaction between primary cilia and the ECM. ECM is a multiplicate well-organized three-dimensional architectural network. It regulates cellular survival, adhesion, growth, and differentiation by operating as communication liaisons between the cells [31]. Melanocyte loss in vitiligo can be caused by defective adhesion to ECM [32,33]. Therefore, it is worthwhile to examine another known cilia-associated signaling, cilia–ECM interaction. In our study, we observed reduced levels of paxillin, FAK, and integrin β1 in melanocytes following OFD1 knockdown (Figure 5a) and the lesional epidermis of vitiligo patients (Figure 5c). Paxillin is a focal-contact-associated protein found in the melanocytes [34]. Although the binding between paxillin and OFD1 has not been previously identified in melanocytes, our immunoprecipitation results (Figure 5b) demonstrate this interaction. The decreased phosphorylation of paxillin at residue 118 following *OFD1* knockdown, accompanied by increased phosphorylation upon *OFD1* overexpression (Figure 5d) and the co-localized upregulation of phosphorylated paxillin by *OFD1* overexpression (Figure 5e), suggest a role for OFD1 in focal adhesion by regulating dynamic adhesion behavior through the tyrosine phosphorylation of paxillin [24].

Dysregulation of cilia–ECM interactions, which has been well documented in polycystic kidney disease [35], is supposed to cause ciliopathies as per that of other cilia-associated signaling pathways. Therefore, the role of cilia–ECM interaction in OFD1-associated melanocyte apoptosis may be insufficient to explain the lack of a direct association between increased melanocyte apoptosis and impaired ciliogenesis in *IFT88-* or *RPGRIP1L*-knockdown melanocytes. Moreover, it is worth noting that vitiligo is not typically a clinical manifestation associated with OFD1 syndrome, a lethal X-linked dominant inherited disease in males, exemplifying a ciliopathy [15]. It is important to highlight that all mutations identified in patients with OFD1 syndrome are located in amino acid residues before residue 631 [36,37,38]. This led us to hypothesize that the OFD1 domain responsible for binding to paxillin may differ from the one involved in ciliopathy. Our results from the GST pull-down assay showed that amino acid residues after 601 of OFD1, but not residues 3-601, interact with the N-terminal-containing LD domains of paxillin (Figure 6a). Furthermore, in vitro staining of melanocytes transfected with chimeric FLAG-*OFD1* demonstrated that the OFD1 amino acid residues after 601 were not involved in ciliogenesis (Figure 6b), supporting our hypothesis.

Paxillin, acting as a scaffold and signaling protein, interacts with multiple proteins, including FAK and integrin β1 [25,39]. FAK, a nonreceptor tyrosine kinase, localizes to focal adhesion complexes, which link the actin cytoskeleton to the ECM. In the skin, the ECM is divided into two types of compartments, interstitial dermal matrix and basement membrane. The interstitial dermal matrix is mainly composed of collagens and elastic fibers. The basement membrane is rich in laminin and type IV collagen [31]. Integrins, particularly integrin ɑ2β1, are well-known cell adhesion receptors involved in the attachment of normal human melanocytes to laminin and fibronectin [40]. Fibronectin is a major component of interstitial dermal matrix [41]. The detachment of melanocytes from fibronectin or type IV collagen has been proposed as one of the apoptotic mechanisms of melanocytes in vitiligo [42,43,44,45,46]. In our study, we found that *OFD1* knockdown inhibited melanocyte adhesion to fibronectin and type IV collagen, and this effect was restored by *PXN* overexpression (Figure 7a). The number of mCherry-positive cells increased following mCherry-fused *PXN* overexpression in melanocytes with *OFD1* knockdown (Figure 7b). Moreover, the reduced levels of FAK and integrin β1 were restored by *PXN* overexpression (Figure 7c). These results suggest that OFD1 knockdown induced melanocyte apoptosis by inhibiting cell adhesion via the downregulation of paxillin, resulting in reduced levels of FAK and integrin β1. The fact that *PXN* knockdown also inhibited melanocyte adhesion (Figure 7d), without affecting melanocyte adhesion to fibronectin following *IFT88* knockdown (Figure 7e), highlights the unique role of *OFD1* in melanocyte survival through paxillin-mediated attachment to the ECM, including the basement membrane.

In summary, our findings suggest that OFD1 downregulation induces melanocyte apoptosis and vitiligo, independent of impaired ciliogenesis, by reducing melanocyte adhesion to the ECM through paxillin.

## 4. Materials and Methods

### 4.1. Patients

The study included sixteen patients diagnosed with vitiligo (8 men and 8 women) aged between 6 and 54 years (mean 23.1 years) (Table S1), along with four healthy individuals (2 men and 2 women aged between 31 and 52 years, mean 44.3 years). The patient cohort displayed clinical heterogeneity, encompassing various types, activity levels, and treatment regimens. The Institutional Review Board of Dongguk University Ilsan Hospital approved this study (approval No. 2012-69, 2017-56). This study adhered to the principles outlined in the Declaration of Helsinki. Lesional and nonlesional skin specimens were obtained from the roof of the suction blister in eight patients for real-time PCR and were biopsied in another eight patients for immunofluorescence microscopy, following written informed consent from each volunteer.

### 4.2. Normal Human Skin Keratinocyte and Melanocyte Culture

Human epidermal keratinocytes and melanocytes were sourced from Gibco (Thermo Fisher Scientific, Waltham, MA, USA). The keratinocytes were suspended in EpiLife medium (Thermo Fisher Scientific) supplemented with bovine pituitary extract (BPE, 0.2%), recombinant human insulin-like growth factor-1 (rhIGF-1, 0.01 μg/mL), hydrocortisone (0.18 μg/mL), human epidermal growth factor (0.2 ng/mL), and bovine transferrin (BT, 5 μg/mL) (Thermo Fisher Scientific). The melanocytes were suspended in Medium 254 (Thermo Fisher Scientific) supplemented with BPE (0.2%), fetal bovine serum (0.5%), rhIGF-1 (0.01 μg/mL), hydrocortisone (0.18 μg/mL), basic fibroblast growth factor (3 ng/mL), BT (5 μg/mL), heparin (3 μg/mL), and phorbol 12-myristate 13-acetate (10 ng/m) (Thermo Fisher Scientific). Keratinocytes and melanocytes from passages 3 to 6 and 8 to 20, respectively, were utilized for these experiments.

### 4.3. Knockdown of OFD1, IFT88, or RIGRIP1L and Overexpression of OFD1 or PXN

Melanocytes (2 × 10^5^/well) and keratinocytes (1.5 × 10^5^/well) were seeded into six-well plates and incubated for 24 h. The cells were transfected with 25 nM CRISPR-CAS9 sgRNA for human *OFD1*, *IFT88*, *RPGRIP1L*, *PXN*, or a negative control sgRNA (Integrated DNA Technologies, San Diego, CA, USA) using CRISPRMAX transfection reagent (Thermo Fisher Scientific). The mCherry vector (Addgene, Cambridge, MA, USA) containing *OFD1* or *PXN* was transfected into cells using Lipofectamine 2000 (Thermo Fisher Scientific). Cells were used for experiments at 48 h post-transfection. All collected cells were subjected to Western blot analysis, immunohistochemistry, confocal microscopy, immunoprecipitation, and glutathione-S-transferase (GST) pull-down assays.

### 4.4. Real-Time PCR

The quantification of target mRNA levels was performed using a LightCycler Real-Time PCR Machine (Roche, Penzberg, Germany). The relative amount of OFD1 (NM_001330209) mRNAs was calculated as the ratio of each target relative to glyceraldehyde 3-phosphate dehydrogenase (GAPDH, NM_001357943). Primer sequences used for real-time PCR were as follows: OFD1, 5′-gaatctgcagggaacatgc-3′ (forward) and 5′-gcgtccacatgagacatatcc-3′ (reverse); GAPDH 5′-tccactggcgtcttcacc-3′ (forward) and 5′-ggcagagatgatgacccttt-3′ (reverse).

### 4.5. Western Blot Analysis

Equal amounts of extracted proteins (20 μg) were resolved and transferred to nitrocellulose membranes, which were incubated with antibodies to OFD1 (Novus Biologicals, Centennial, CO, USA), paxillin, FAK, PARP, BAD, BAX, WNT3a (Santa Cruz Biotechnology, Santa Cruz, CA, USA), PTCH1, GLI1, Smo, BCL2, p-BAD, GSK-3β, β-catenin, cleaved caspase-3 (Cell Signaling Technology, Beverly, MA, USA), integrin β1 (Bethyl Laboratories, Montgomery, TX, USA), and β-actin (Sigma-Aldrich, St. Louis, MO, USA). Following incubation with the appropriate anti-rabbit or anti-mouse horseradish peroxidase-conjugated antibody (Santa Cruz Biotechnology), enhanced chemiluminescence solution (Thermo Fisher) was added, and signals were captured using an image reader (LAS-3000; Fuji Photo Film, Tokyo, Japan). The protein bands were analyzed by densitometry.

### 4.6. Immunoprecipitation

The cells were solubilized with lysis buffer and centrifuged at 10,000× *g* for 10 min at 4 °C. Supernatants of cell lysates were incubated with anti-OFD1 or antipaxillin antibody and resin in the Pierce™ Direct IP kit (Thermo Fisher Scientific) at 4 °C. Eluted resin-bound proteins were analyzed by immunoblotting with antipaxillin, anti-FAK, or anti-OFD1 antibody.

### 4.7. Immunofluorescent and Confocal Microscopy

Sections were deparaffinized, rehydrated, and preincubated with 3% bovine serum albumin. These sections were sequentially incubated with anti-OFD1, anti-c-KIT (1:200 dilution; DB Biotech, Kosice, Slovakia) or antipaxillin antibodies. After staining with Alexa Fluor^®^ 488-conjugated goat anti-rabbit IgG and/or Alexa Fluor^®^ 594-conjugated goat anti-mouse IgG (1:200 dilution; Molecular Probes, Eugene, OR, USA), the nuclei were counterstained with Hoechst 33258 (Sigma-Aldrich). The cultured cells were fixed in 2% paraformaldehyde and double stained with antiacetylated α-tubulin or anti-FLAG (1:200 dilution; Origene Technologies, Rockville, MD, USA), as well as anti-OFD1, anti-IFT88, anti-RPGRIP1L, anti-p-paxillin, or anti-ARL13B antibodies (1:200 dilution). Subsequently, the cells were stained with Alexa Fluor^®^ 594-conjugated goat anti-mouse IgG and Alexa Fluor^®^ 488-conjugated goat anti-rabbit IgG, and nuclei were counterstained with Hoechst 33258. Fluorescent images were evaluated using an image analysis system (Dp Manager 2.1; Olympus Optical Co., Tokyo, Japan) and Wright Cell Imaging Facility ImageJ software version 1.54d (https://imagej.net/ij/download.html accessed on 26 March 2023). Confocal images were obtained using the EZ-C1 software 3.8 (EZ-C1; Nikon, Tokyo, Japan) and analyzed using NIS-Elements AR 3.2 (Nikon Instruments, Melville, NY, USA).

### 4.8. Plasmid Construction

OFD1 fragments carrying one to six coiled-coil domains (1–5) were amplified by PCR using full-length human *OFD1* cDNA (residues 1–1012, Origene Technologies) as the template. The amplified fragments were subcloned into the bacterial expression vector pGEX-4T-1 (GE Healthcare, Chicago, IL, USA) with the N-terminal GST [24]. All constructs were verified by DNA sequencing.

### 4.9. GST Pull-Down Assay

*Escherichia coli* was cultured until the OD600 nm reached 0.6–0.8 and then induced for 3 h with 0.1 mM isopropyl thiogalactoside at 37 °C (the OD600 nm reached 1). The lysates were applied to equilibrated glutathione columns in the Pierce™ GST Protein Interaction Pull-Down Kit (Thermo Fisher Scientific) for 2 h at 4 °C. The 293T cell extracts transfected with *PXN* were incubated with the proteins bound to glutathione-sepharose beads overnight at 4 °C. The following day, the beads were washed with the buffer four times and resuspended in the protein sample buffer for SDS-PAGE and Western blot analysis.

### 4.10. Cell Viability Test

The cells were stained with 3-(4,5-dimethylthiazol-2yl)-2,5-diphenyltetrazolium bromide (MTT) for 4 h. The precipitated formazan was dissolved in DMSO and the optical density was measured at 570 nm with background subtraction at 630 nm using a microplate reader (Spark; TECAN, Männedorf, Switzerland).

### 4.11. Flow Cytometry Analysis

Cells were harvested 24 h after *OFD1* knockdown and labeled using the Dead Cell Apoptosis Kit with Annexin V Alexa Fluor^TM^ 488 and Propidium Iodide (Thermo Scientific). Labeled cells were analyzed with a Cytomics™ FC500 Flow Cytometry (Beckman Coulter, Indianapolis, IN, USA).

### 4.12. Cell Adhesion Assay

We coated 24-well culture plates with 30 μg/mL type IV collagen or fibronectin (Corning Life Sciences, Bedford, MA, USA). Melanocytes (2 × 10^4^) with or without *OFD1* knockdown or *PXN* overexpression were plated and allowed to adhere to the coated surface at 37 °C for 30 min. After fixation in 4% paraformaldehyde and staining of the nuclei with Hoechst 33258, the number of stained cells in a fixed area was counted under a standard light microscope (DM LB microscope; Leica Microsystems, Wetzlar, Germany). A similar assay was also conducted using melanocytes with or without the silenced *PXN* and *IFT88* genes.

### 4.13. Statistical Analysis

Statistical analyses were performed using GraphPad Prism 8 software (GraphPad Software, La Jolla, CA, USA). A *p*-value of <0.05 was considered significant and indicated by * *p* < 0.05, ** *p* < 0.01, and *** *p* < 0.001. For comparisons between the two groups, a two-tailed Student’s unpaired *t*-test was used for parametrical data. One- or two-way analysis of variance (ANOVA) was used for comparisons among multiple groups and parameters. Means ± SD were calculated for in vitro experimental data, while differences between nonlesions and lesions in human sample data were assessed by the Mann–Whitney test and expressed as mean ± standard error of the mean (SEM).

## 5. Patents

Patent information is included in the Section 4 of the original manuscript.

## Data Availability

Data are contained within the article.

## Supplementary Materials

The following supporting information can be downloaded at: https://www.mdpi.com/article/10.3390/ijms242417528/s1.
