# Supplementary material for: Extraciliary OFD1 Is Involved in Melanocyte Survival through Cell Adhesion to ECM via Paxillin
Source: Int J Mol Sci. 2023 Dec 15;24(24):17528. doi: 10.3390/ijms242417528 (PMC10743763; doi:10.3390/ijms242417528)
Supplement: Supplementary file 1 [file ijms-24-17528-s001.zip › Figure S1.pdf]

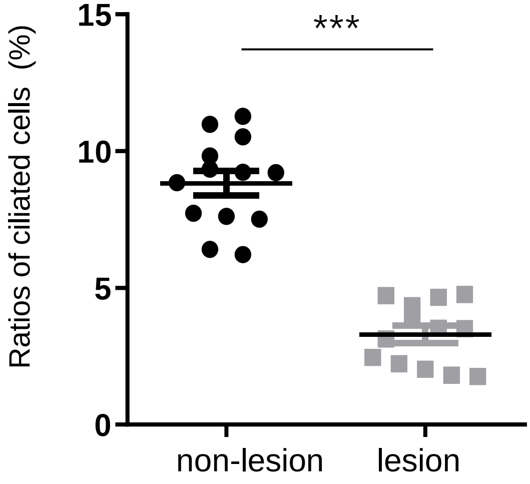

Figure S1. Confocal microscopy using anti-Arl13b and anti-γ-tubulin antibodies was performed in biopsied skin samples derived from five vitiligo patients with OFD1 downregulation. The number of ciliated cells was counted in two to three randomly selected high-power fields in each specimen (total 13).
