# Supplementary material for: Extraciliary OFD1 Is Involved in Melanocyte Survival through Cell Adhesion to ECM via Paxillin
Source: Int J Mol Sci. 2023 Dec 15;24(24):17528. doi: 10.3390/ijms242417528 (PMC10743763; doi:10.3390/ijms242417528)
Supplement: Supplementary file 1 [file ijms-24-17528-s001.zip › Table S1.pdf]

Table S1. Patient demographics.

No	Sex /Age	Duration	Type	Activity	Previous Treatment	Lesional Location and Sampling Sites
1	M/14	2 yrs	Non-segmental	Stable	Steroid+Excimer	Face, upper and lower extremities, dorsa of feet
2	M/15	Several yrs	Non-segmental	Active	None	Face, upper extremities, back
3	M/16	6 mos	Non-segmental	Stable	Steroid+NB-UVB	Trunk, inguinal, lower extremities
4	M/20	2 yrs	Non-segmental	Active	Steroid	Scalp, forehead, nose, perioral, neck, anterior chest, both upper extremities
5	M/31	9 yrs	Non-segmental	Active	Steroid+NB-UVB	Wrists, dorsa of feet, anterior chest, abdomen
6	F/18	1 yrs	Non-segmental	Active	None	Arms, trunk
7	F/18	>5 yrs	Non-segmental	Active	Steroid+NB-UVB	Hands, wrists, anterior chest, abdomen, inguinal
8	F/54	2 yrs	Non-segmental	Active	None	Anterior chest, abdomen, both groins
9	M/18	4 yrs	Segmental	Stable	Steroid+NB-UVB	Lt forearm
10	F/16	a few yrs	Segmental	Stable	Steroid+NB-UVB	Lt breast, anterior chest
11	F/51	a few yrs	Segmental	Active	None	Rt. Buttock, posterior thigh
12	F/52	2 yrs	Segmental	Stable	Tacrolimus+NB-UVB	Rt eyelid, nose, forehead
13	F/8	4 yrs	Mixed	Stable	Steroid+NB-UVB	Rt upper lip, chin, neck, scalp
14	M/15	>10 yrs	Mixed	Stable	None	Rt forehead
15	M/18	a few yrs	Localized	Stable	Steroid+Excimer	Rt inner thigh
16	F/9	5 yrs	Localized	Stable	Steroid+NB-UVB	Posterior neck, anterior chest

yrs: years, mos; months, steroid: topical steroid, tacrolimus: topical tacrolimus, Lt: left, Rt: right Sampling site of each patient was underlined.
